# Physiological Assessment with iFR prior to FFR Measurement in Left Main Disease

**DOI:** 10.1007/s12928-024-00989-4

**Published:** 2024-04-20

**Authors:** Takayuki Warisawa, Christopher M. Cook, Yousif Ahmad, James P. Howard, Henry Seligman, Christopher Rajkumar, Takumi Toya, Shunichi Doi, Akihiro Nakajima, Masafumi Nakayama, Rafael Vera-Urquiza, Sonoka Yuasa, Takao Sato, Yuetsu Kikuta, Yoshiaki Kawase, Hidetaka Nishina, Rasha Al-Lamee, Sayan Sen, Amir Lerman, Hitoshi Matsuo, Yoshihiro J. Akashi, Javier Escaned, Justin E. Davies

**Affiliations:** 1 Department of Cardiology, St. Marianna University School of Medicine, 2-16-1 Sugao, Kawasaki, 216-8511 Japan; 2 Department of Cardiology, NTT Medical Center Tokyo, Tokyo, Japan; 3 National Heart and Lung Institute, Imperial College London, London, UK; 4 The Essex Cardiothroacic Centre, Essex, UK; 5 Anglia Ruskin University, Essex, UK; 6 Cardiovascular Medicine, Yale School of Medicine, New Haven, USA; 7 Cardiovascular Science, Hammersmith Hospital, Imperial College Healthcare NHS Trust, London, UK; 8 Guys and St, Royal Brompton and Harefield Hospitals, Thomas NHS Foundation Trust, London, UK; 9 Department of Cardiology, National Defense Medical College, Tokorozawa, Japan; 10 Department of Cardiovascular Medicine, Mayo Clinic, Rochester, USA; 11 Department of Cardiovascular Medicine, New Tokyo Hospital, Matsudo, Japan; 12 Department of Cardiology, Tokyo D Tower Hospital, Tokyo, Japan; 13 Cardiovascular Center, Toda Central General Hospital, Toda, Japan; 14 Hospital Clinico San Carlos IDISSC, Complutense University of Madrid, Madrid, Spain; 15 Department of Cardiology, Tachikawa General Hospital, Nagaoka, Japan; 16 Division of Cardiology, Fukuyama Cardiovascular Hospital, Fukuyama, Japan; 17 Department of Cardiovascular Medicine, Gifu Heart Center, Gifu, Japan; 18 Department of Cardiology, Tsukuba Medical Center Hospital, Tsukuba, Japan

**Keywords:** Coronary physiology, Deferral, Fractional flow reserve, Instantaneous wave-free ratio, Left main coronary artery disease, Revascularization

## Abstract

Despite guideline-based recommendation of the interchangeable use of instantaneous wave-free ratio (iFR) and fractional flow reserve (FFR) to guide revascularization decision-making, iFR/FFR could demonstrate different physiological or clinical outcomes in some specific patient or lesion subsets. Therefore, we sought to investigate the impact of difference between iFR and FFR-guided revascularization decision-making on clinical outcomes in patients with left main disease (LMD). In this international multicenter registry of LMD with physiological interrogation, we identified 275 patients in whom physiological assessment was performed with both iFR/FFR. Major adverse cardiovascular event (MACE) was defined as a composite of death, non-fatal myocardial infarction, and ischemia-driven target lesion revascularization. The receiver-operating characteristic analysis was performed for both iFR/FFR to predict MACE in respective patients in whom revascularization was deferred and performed. In 153 patients of revascularization deferral, MACE occurred in 17.0% patients. The optimal cut-off values of iFR and FFR to predict MACE were 0.88 (specificity:0.74; sensitivity:0.65) and 0.76 (specificity:0.81; sensitivity:0.46), respectively. The area under the curve (AUC) was significantly higher for iFR than FFR (0.74; 95%CI 0.62–0.85 vs. 0.62; 95%CI 0.48–0.75; *p* = 0.012). In 122 patients of coronary revascularization, MACE occurred in 13.1% patients. The optimal cut-off values of iFR and FFR were 0.92 (specificity:0.93; sensitivity:0.25) and 0.81 (specificity:0.047; sensitivity:1.00), respectively. The AUCs were not significantly different between iFR and FFR (0.57; 95%CI 0.40–0.73 vs. 0.46; 95%CI 0.31–0.61; *p* = 0.43). While neither baseline iFR nor FFR was predictive of MACE in patients in whom revascularization was performed, iFR-guided deferral seemed to be safer than FFR-guided deferral.

**Graphical abstract:**

Impact of Physiological Assessment with iFR and FFR on Clinical Outcomes of Patients with LMD. In the present study, physiological assessment, both with iFR and FFR, provided a high predictability of adverse cardiovascular event in LMD patients with revascularization deferral. Furthermore, the iFR-guided deferral strategy was safer as compared to FFR. Conversely, in patients in whom revascularization was performed for LMD, neither iFR nor FFR was predictive of cardiovascular event. AUC: area under the curve; FFR: fractional flow reserve; iFR: instantaneous wave-free ratio; LMD: left main coronary artery disease.

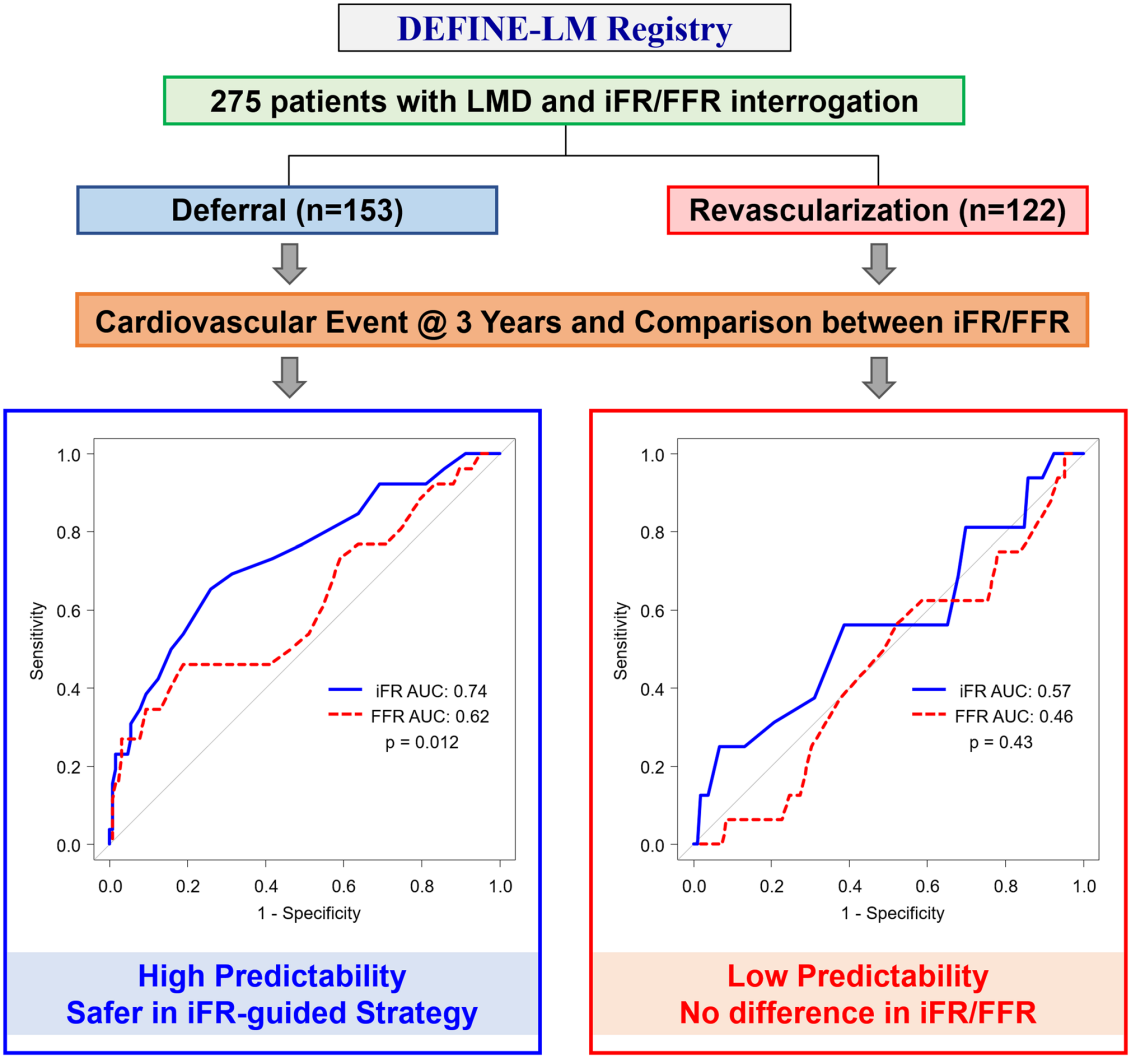

**Supplementary Information:**

The online version contains supplementary material available at 10.1007/s12928-024-00989-4.

## Introduction

Left main coronary artery disease (LMD) has been considered potentially fatal if not treated with coronary revascularization. Ealy reports from decades ago demonstrated an extremely poor prognosis in patients with LMD treated with medical therapy alone, showing a 5 year survival of less than 50% [1, 2]. Despite advances in the contemporary medical therapy of stable coronary artery disease (CAD), a prognosis in patients with physiologically significant LMD is recently reported to still remain suboptimal with rates of adverse cardiovascular events of approximately 30% at 4 years [3]. Considering such a high-risk disease entity, diagnostic guidelines recommend ruling out LMD as a first step of stable CAD [4, 5].

For stable CAD, international treatment guidelines recommend the *interchangeable* use of instantaneous wave-free ratio (iFR) and fractional flow reserve (FFR) to guide revascularization decision-making [5–7]. However, it is reported that in some specific patient or lesion subsets, iFR and FFR can demonstrate different physiological outcomes. Specifically, discordant results can be observed in ∼20% of cases when the respective cut-off values (0.89 for iFR and 0.80 for FFR) are adopted [8–12]. Furthermore, iFR/FFR discordance is known to occur more frequently in LM stem and proximal left anterior descending artery (LAD) disease, and some in the physiological community have cautioned against the use of non-hyperemic pressure ratios (NHPR) in such anatomies [12].

Conversely, recent studies from the DEFINE-LM registry and the iLITRO-EPIC07 registry demonstrated the apparent safety of LM-revascularization deferral based on iFR, by demonstrating similar adverse cardiovascular outcomes as compared with patients in whom LM-revascularization was performed [13, 14]. However, the direct comparison between NHPR and FFR in LMD has not yet been reported.

Therefore, in this present study, we sought to investigate the impact of difference between iFR and FFR-guided revascularization decision-making on clinical outcomes in patients with stable CAD and LMD, using data from the DEFINE-LM registry.

## Methods

### Study population

As described previously [13], the DEFINE-LM (deferral of coronary revascularization based on instantaneous wave-free ratio evaluation for left main coronary artery disease) registry is an international multicenter registry, comprising patients of LMD between October 2012 to October 2018 at 10 cardiac centers in Europe, the U.S.A. and Japan. Consecutive patients were included when the following criteria were met: patients with stable angina; LMD of 40–70% on visual angiographic assessment; and iFR interrogation for LMD. Exclusion criteria were as follows: previous coronary artery bypass grafting (CABG) or previous percutaneous coronary intervention (PCI) for LMD; severe valvular pathology; and any type of non-ischemic cardiomyopathy.

**Fig. 1:**
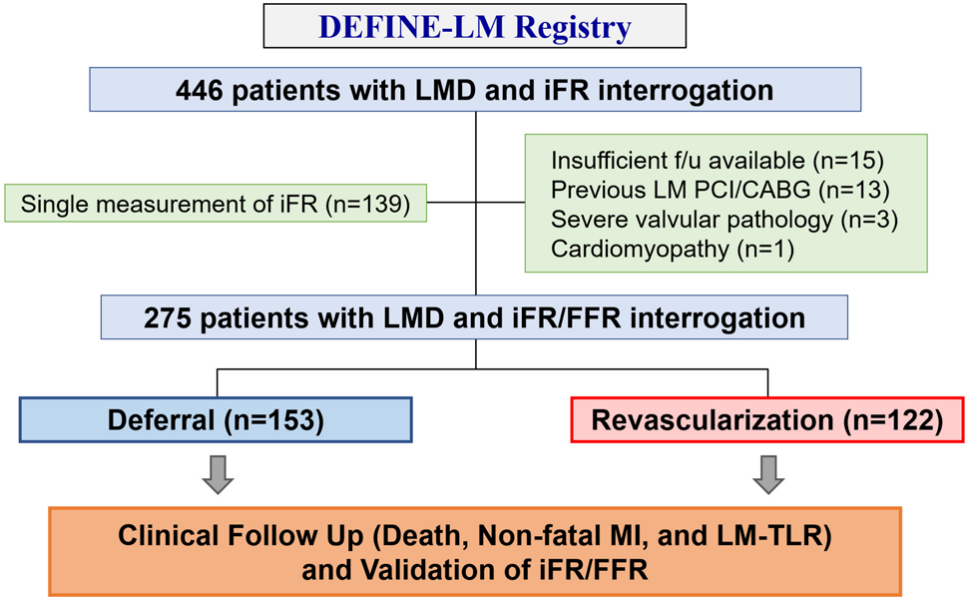
Study Flow. Consecutive cases of de-novo stable left main disease and physiological assessment using both iFR and FFR were analyzed. *CABG*: coronary artery bypass grafting; *FFR*: fractional flow reserve; *iFR*: instantaneous wave-free ratio; *LMD*: left main coronary artery disease; *MI*: myocardial infarction; *PCI*: percutaneous coronary intervention; *LM-TLR*; target lesion revascularization of LMD

From this dataset, we identified patients in whom physiological assessment was concurrently also performed with FFR. We assessed the long-term clinical outcomes in patients with stable de-novo LMD of intermediate angiographic severity in whom revascularization was deferred and performed, respectively. The study flow diagram is shown in Fig. 1. All patients provided written informed consent. This study was approved by the local ethical committees at each participating center and was conducted according to the principles of the Declaration of Helsinki.

### iFR/FFR measurement

The detail of the physiological assessment of LMD is described elsewhere [13]. Specifically, iFR/FFR were measured at the distal point of the LM segment either in the LAD or left circumflex artery (LCx). The measured indices are expressed as iFR_LM_ and FFR_LM_, respectively in this study. If the bifurcation lesion involved an ostial LAD or LCx, it was also considered as LM segment. If intracoronary pressure was measured in both the LAD and LCx (such as in the case of a bifurcation lesion), the lower value was used to assess physiological significance. When further downstream disease was present in the LAD or LCx, the wire was placed either in the non-diseased artery or proximal to the first angiographical stenosis. In the actual measurement of iFR/FFR, disengagement of the guiding catheter from the left coronary artery orifice was appropriately confirmed as recommended [15], considering that some cases may have had ostial disease of the LM stem. The decision to perform FFR assessment following iFR measurement was left to the operator’s discretion. In measuring FFR, hyperemia was induced by intravenous adenosine infusion (140–160 μg/kg/min). Routine cut-off values of hemodynamic significance were used (iFR ≤ 0.89 and FFR ≤ 0.80).

### Comparative assessment of clinical outcomes between iFR and FFR

The pre-defined clinical outcomes in this registry-based study were evaluated as the rate of major adverse cardiovascular events (MACE) over follow-up. MACE was defined as a composite of all-cause death, non-fatal myocardial infarction (MI), and ischemia-driven target lesion revascularization of LMD (LM-TLR). MI included both ST-segment elevation MI and non-ST-segment elevation MI. LM-TLR was recorded as a MACE when it was not the index procedure and was not identified at the time of the index procedure as a staged procedure to occur within 60 days. Patients were followed up for clinical visits at each participating center. When needed, patients or their general practitioners/family doctors were contacted for additional confirmatory clinical information. Comparison of predictability for MACE between iFR_LM_ and FFR_LM_ was performed in respective patients in whom revascularization was deferred and performed, which was the primary endpoint of the present study.

### Statistical analysis

Categorical data are expressed as numbers and percentages. Continuous variables are expressed as mean and (±) standard deviation or as median accompanied by interquartile range (IQR) as appropriate. Continuous variables were compared with Student t or Mann-Whitney U tests, and categorical variables with chi-square or Fisher exact tests, as appropriate. The dependent variable in the analysis was time to initial events during follow-up. Kaplan-Meier curves for MACE-free survival were constructed and compared between deferral and revascularization groups through the log-rank test, while relative differences were summarized by HRs (hazard ratios) and 95% CIs (confidence intervals) from Cox regression models. Where one arm showed no events, log-rank p values for those outcomes were provided alone, without HRs and associated CIs. The receiver-operating characteristic analysis was performed for both iFR_LM_ and FFR_LM_ to predict MACE in respective patients in whom revascularization was deferred and performed. To determine the optimal cut-off value for iFR_LM_ and FFR_LM_, the point on the receiver-operating characteristic curve that is closest to the (0,1) point, representing perfect sensitivity and specificity balance was identified. The area under the curve (AUC) was compared using the DeLong test between iFR_LM_ and FFR_LM_ in patients with revascularization deferral and coronary revascularization, respectively.

All probability values were two-sided, and p values < 0.05 were considered statistically significant. All the statistical analysis was performed using R version 4.1.2 (R Foundation for Statistical Computing, Vienna, Austria).

## Results

### Overall study population

A total of 275 patients were included in this analysis (Fig. 1). Mean age was 67.9 ± 10.2 years (78.2% male). Mean SYNTAX (Synergy Between PCI With Taxus and Cardiac Surgery) score was 19.6 ± 8.7 and mean percent diameter stenosis was 45.2 ± 14.2%. The median iFR_LM_ and FFR_LM_ values were 0.88 (IQR: 0.82 to 0.92) and 0.77 (IQR: 0.70 to 0.84), respectively. Coronary revascularization was deferred for 153 [55.6%] patients and performed for 122 [44.4%] patients, respectively (PCI: *n* = 67 [54.9%]; CABG: *n* = 55 [45.1%]). Full description of the baseline and lesion characteristics is provided in Supplemental Table S1.

For all patients, guideline-directed medical therapy was initiated as per contemporary clinical practice in each participating center. In the PCI arm, latest generation drug-eluting stents were used under the guidance of intracoronary imaging modalities in *all* cases (single stenting to LM-LAD: 62/67 [92.5%]; two stent technique: 5/67 [7.5%]), while internal mammary artery grafting was used for the LAD in *all* cases within the CABG arm.

### Baseline and lesion characteristics in the deferred vs. revascularized groups

While the frequency of cardiovascular risk factors was not different between two groups, the deferral group included older (69.5 ± 10.1 vs. 66.0 ± 10.1; *p* = 0.0046) and more female (28.1% vs. 13.9%; *p* = 0.0074) patients. Regarding vessel and lesion characteristics, in the revascularization group, lesion complexity and stenosis severity were significantly greater than those in the deferral group. Specifically, the revascularization group showed higher frequencies of LAD involvement and multi-vessel disease, resulting in significantly higher SYNTAX scores (18.1 ± 9.1 vs. 21.4 ± 7.9; *p* = 0.0016). Angiographic and physiologic stenosis severities were also greater in the revascularization group: diameter stenosis: 41.8 ± 13.5% vs. 49.5 ± 14.0%; *p* < 0.001; minimum lumen diameter: 2.20 ± 0.67mm vs. 1.85 ± 0.62mm; *p* < 0.001; lesion length: 12.0 ± 6.4mm vs. 15.0 ± 8.1mm; p < 0.001; iFR_LM_: 0.91 [0.88–0.94] vs. 0.85 [0.76–0.88]; *p* < 0.001; FFR_LM_: 0.82 [0.77–0.87] vs. 0.71 [0.65–0.76]; *p* < 0.001). Full description of the baseline and lesion characteristics in each treatment strategy is provided in Table 1.

**Table 1:** Patient and Lesion Characteristics according to the Treatment Strategy

	Deferral (*n* = 153)	Revascularization (*n* = 122)	p value
*Patient characteristics*			
Age, yrs	69.5 ± 10.1	66.0 ± 10.1	0.0046
Male	110 (71.9)	105 (86.1)	0.0074
Hypertension	117 (76.5)	91 (74.6)	0.83
Dyslipidemia	99 (64.7)	86 (70.5)	0.38
Diabetes mellitus	52 (34.0)	50 (41.0)	0.29
Chronic kidney disease	28 (18.3)	35 (28.7)	0.059
Current smoker	57 (37.3)	34 (27.9)	0.13
Family history of CAD	27 (17.6)	17 (13.9)	0.50
Previous MI	44 (28.8)	33 (27.0)	0.86
*Vessel and lesion characteristics*			
*Left main lesion type*			
Ostial type	32 (20.9)	36 (29.5)	0.13
Mid type	31 (20.3)	31 (25.4)	0.38
Distal type	118 (77.1)	102 (83.6)	0.24
*Other diseased vessels*			
No. of diseased vessels			0.023
0	31 (20.3)	16 (13.1)	
1	50 (32.7)	26 (21.3)	
2	43 (28.1)	50 (41.0)	
3	29 (19.0)	30 (24.6)	
LAD	83 (54.2)	93 (76.2)	<0.001
LCx	63 (41.2)	65 (53.3)	0.061
RCA	77 (50.3)	58 (47.5)	0.74
With CTO	16 (10.5)	12 (9.8)	1.00
SYNTAX Score	18.1 ± 9.1	21.4 ± 7.9	0.0016
*Quantitative coronary angiography*			
% Diameter stenosis, %	41.8 ± 13.5	49.5 ± 14.0	<0.001
Minimum lumen diameter, mm	2.20 ± 0.67	1.85 ± 0.62	<0.001
Reference diameter, mm	3.87 ± 0.99	3.70 ± 0.71	0.12
Lesion length, mm	12.0 ± 6.4	15.0 ± 8.1	<0.001
*Physiological stenosis severity*			
iFR_**LM**_	0.91 (0.88–0.94)	0.85 (0.76–0.88)	<0.001
FFR_**LM**_	0.82 (0.77–0.87)	0.71 (0.65–0.76)	<0.001

Values are mean $\pm$ SD, n (%), or median (interquartile range)

*CAD*: coronary artery disease; *CTO*: chronic total occlusion; *FFR*: fractional flow reserve; *iFR*: instantaneous wave-free ratio; *LAD*: left anterior descending artery; *LCx*: left circumflex artery; *MI*: myocardial infarction; *MLD*: minimum lumen diameter; *RCA*: right coronary artery; *SYNTAX*: Synergy Between PCI With Taxus and Cardiac Surgery

### Clinical outcomes

**Fig. 2:**
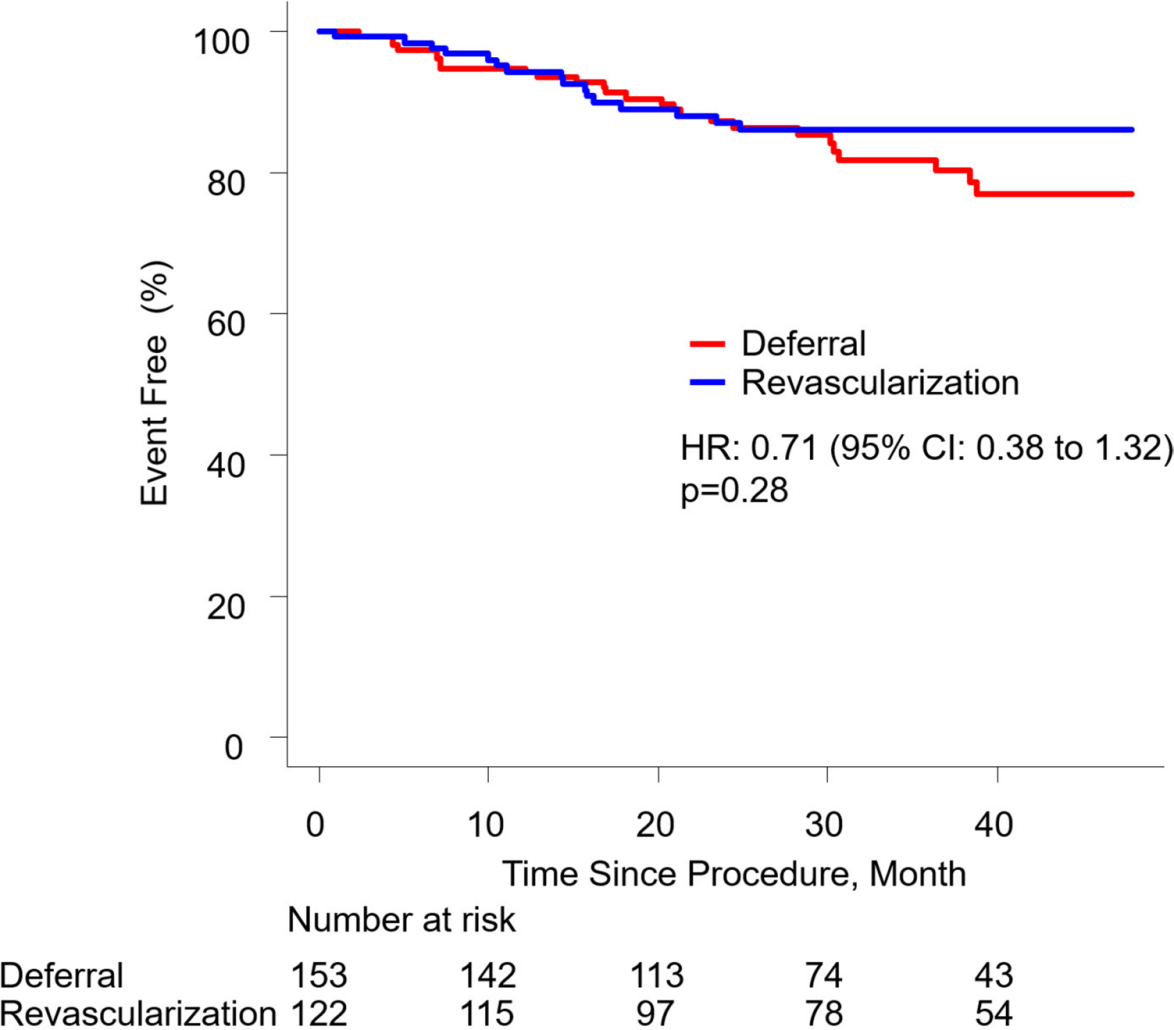
Major Adverse Cardiovascular Events between Deferral vs. Revascularization. Kaplan-Meier event-free curves showing major adverse cardiovascular events in the two groups. *CI*: confidence interval; *HR*: hazard ratio

The median follow-up period was 35 months (IQR: 24 to 45). MACE occurred in 26 patients (17.0%) in the deferred group and 16 patients (13.1%) in the revascularized group. Kaplan-Meier event-free survival estimates at 4 years demonstrated no significant difference between the two groups (HR: 0.71; CI: 0.38 to 1.32; *p* = 0.28) (Fig. 2). For each component of MACE, findings in the deferred and revascularized groups were as follows: all-cause death: 7.2% vs. 2.5% (HR: 0.29; CI 0.08 to 1.05; *p* = 0.06); non-fatal MI: 2.0% vs. 4.1% (HR: 1.86; CI 0.44 to 7.82; *p* = 0.40); and LM-TLR: 9.8% vs. 6.6% (HR: 0.62; CI 0.26 to 1.45; *p* = 0.27), respectively. Specifically, none of the clinical outcomes were statistically different between the deferral and revascularization groups, though the rate of all-cause death was numerically higher in the deferral group. Among the revascularized group, there were no differences in the rates of MACE between PCI and CABG during follow-up (9/67 [13.4%] vs. 7/55 [12.7%]; HR: 1.15; CI 0.43 to 3.11; *p* = 0.78).

### Details of MACE

Detailed description of MACE is provided in Table 2. While there was no LM-MIs in the revascularized group, only LM-MIs were observed in the deferral group. More specifically, in the deferred group, 11 patients died during follow-up, of which 3 cases were considered to be cardiac death. There were 3 non-fatal MIs and 15 LM-TLRs (5 CABG and 10 PCI). Three non-fatal MIs required urgent PCI for LM stenosis and the remaining 12 LM-TLRs were electively performed due to the recurrent angina. In the revascularized group, 3 patients died during follow-up and 2 cases were considered to be cardiac. There were 5 non-fatal MIs, 2 of which were non-LM MIs (both in the right coronary artery) in the PCI arm and 3 of which were due to the acute occlusion of a saphenous vein grafts to either the LCx or the right coronary artery in the CABG arm. Stent thrombosis was not observed in this population. LM-TLR was observed in 8 LM stenosis patients, consisting of 2 cases that underwent PCI in the native LM stenosis because of an occluded left internal mammary artery graft to the LAD and 6 cases that underwent additional PCI for LM in-stent restenosis. One patient died one year after percutaneous TLR for LM in-stent restenosis. The causes of non-cardiac death in each group are summarized in Supplemental Table S2.

**Table 2:** Details of Major Adverse Cardiovascular Events

		Deferral (*n* = 153)	Revascularization (*n* = 122)
Death	Cardiac	3 (2.0)	2 (1.6)
	Non-Cardiac	8 (5.2)	1 (0.82)
MI	LM	3 (2.0)	0 (0.0)
	Non-LM	0 (0.0)	5 (4.1)
LM-TLR	Emergent	3 (2.0)	0 (0.0)
	Elective	12 (7.8)	8 (6.6)

Values are n (%)

*CABG*: coronary artery bypass grafting; *LM*: left main related; *PCI*: percutaneous coronary intervention; *TLR*: target lesion revascularization. Other abbreviation as in Table 1.

### Comparison between iFR and FFR in LMD

**Fig. 3:**
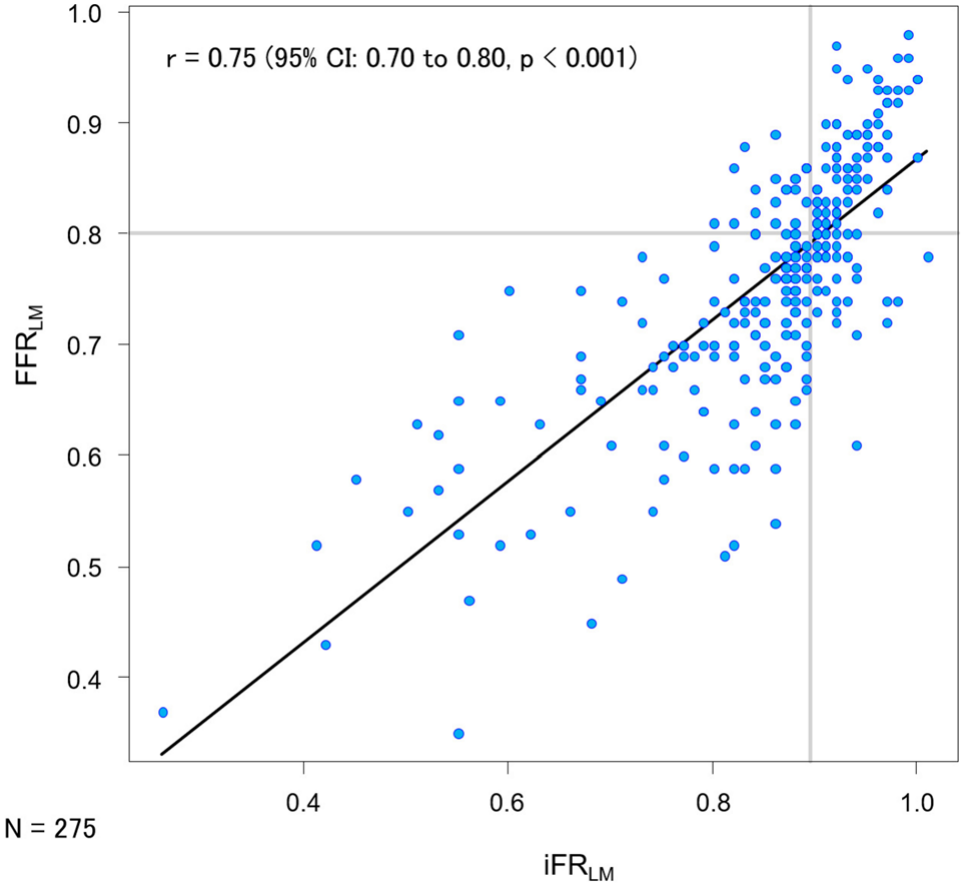
Scatter plot showing the relationship between FFR_LM_ and iFR_LM_ values in LMD. The black line represents the line of best fit. The gray lines represented the respective cut-off values for iFR (≦0.89) and FFR (≦0.80). Abbreviation as in Fig. 1

The relationship between iFR_LM_ and FFR_LM_ values is displayed in Fig. 3 (*r* = 0.75; 95% CI 0.70 to 0.80; *p* < 0.001). Discordant results between iFR_LM_ and FFR_LM_ were observed in 21.1% (58/275) of cases (iFR_LM_ ≤ 0.89 and FFR_LM_ > 0.80: *n* = 20 [7.3%]; iFR_LM_ > 0.89 and FFR_LM_ ≤ 0.80: *n* = 38 [13.8%]). Distributions of iFR_LM_ and FFR_LM_ values according to the treatment allocation are summarized in Supplemental Fig. S1. Of note, in the deferral group (*n* = 153), approximately 40% of cases were deferred in fact despite physiological confirmation of myocardial ischemia: iFR_LM_ ≤ 0.89 or FFR_LM_ ≤ 0.80 (37.9% vs 41.8%, respectively).

**Fig. 4:**
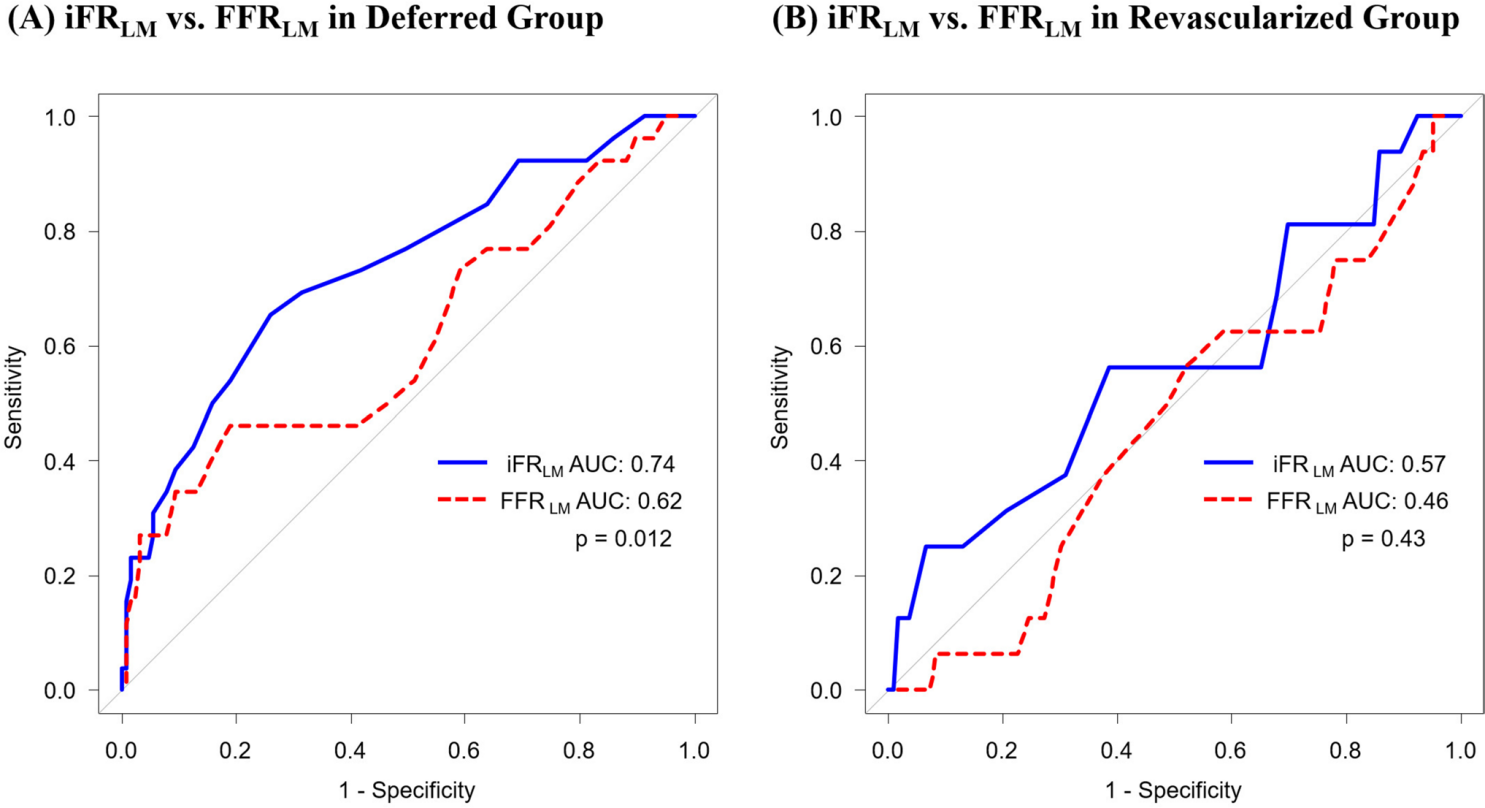
Receiver-Operating Characteristic Curves of iFR and FFR in LMD. Comparison of receiver-operating characteristic curves between iFR_**LM**_ and FFR_**LM**_ in the deferred group (**A**) and revascularized group (**B**). The AUC was significantly higher for iFR_**LM**_ than FFR_**LM**_ in the deferred group while curves were not different in the revascularized group. *AUC*: area under the curve. Other abbreviation as in Fig. 1

In the deferred group, the optimal cut-off values of iFR_LM_ and FFR_LM_ to predict MACE were 0.88 (specificity: 0.74; sensitivity 0.65) and 0.76 (specificity: 0.81; sensitivity: 0.46), respectively. The AUC was significantly higher for iFR_LM_ than FFR_LM_ (0.74 [95% CI 0.62 to 0.85] vs. 0.62 [95% CI 0.48 to 0.75]; *p* = 0.012) (Fig. 4A). Clinical utility of iFR_LM_ was confirmed by the decision curve analysis as well (Supplemental Fig. S2).

In the revascularized group, the optimal cut-off values of iFR_LM_ and FFR_LM_ to predict MACE were 0.92 (specificity: 0.93; sensitivity 0.25) and 0.81 (specificity: 0.047; sensitivity: 1.00), respectively. The AUCs were not significantly different between iFR_LM_ and FFR_LM_ (0.57 [95% CI 0.4 to 0.73] vs. 0.46 [95% CI 0.31 to 0.61]; *p* = 0.43) (Fig. 4B).

**Fig. 5:**
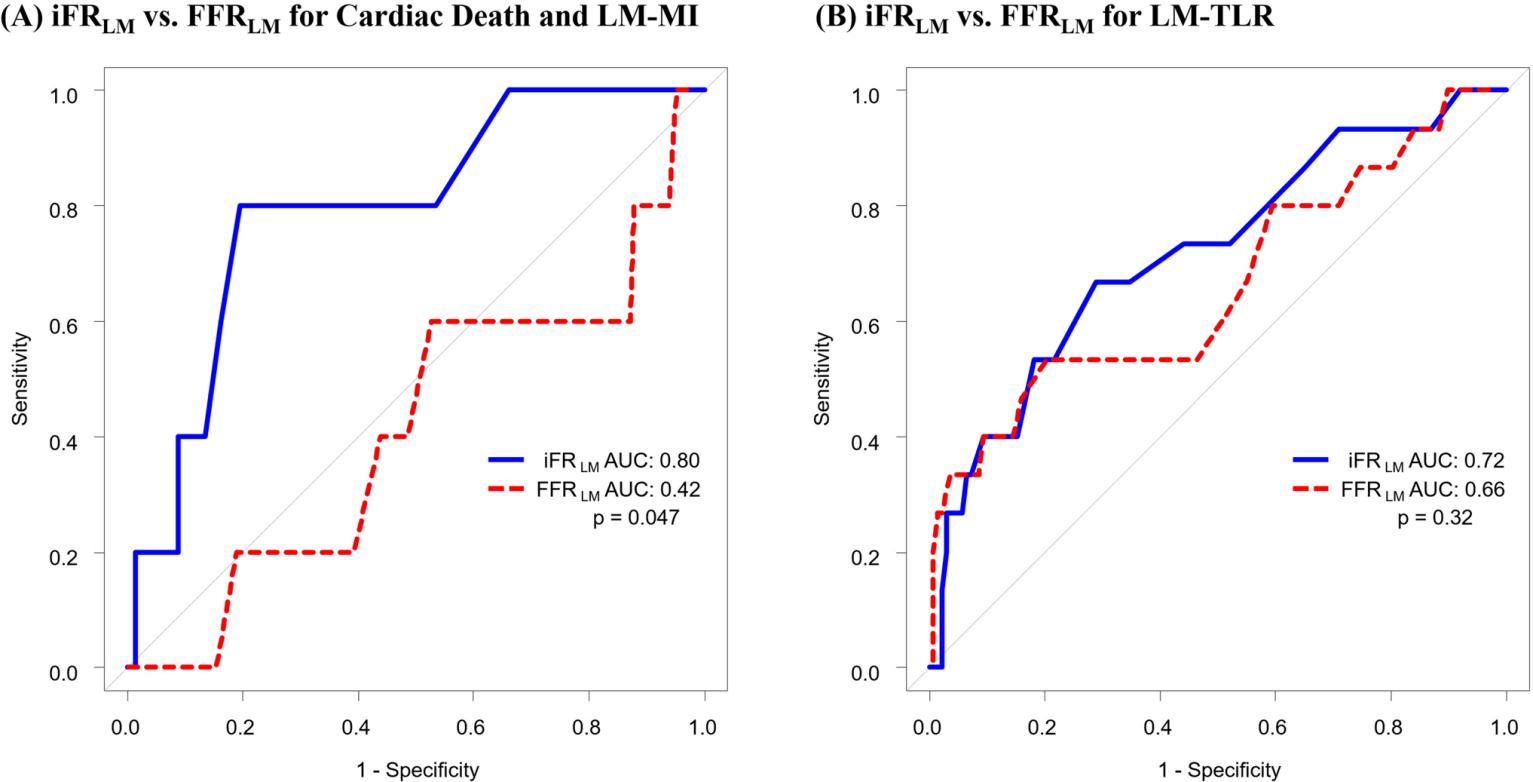
Predictability Comparison between iFR_LM_ and FFR_LM_ for Hard and Non-hard Cardiovascular Events in the Deferral Group. **A** Receiver-operating characteristic curves for cardiac death and LM-MI. **B** Receiver-operating characteristic curves for LM-TLR. The AUC was significantly higher for iFR_**LM**_ than FFR_**LM**_ only for hard cardiovascular events. Abbreviation as in Figs. 1 and 4

Regarding the deferral group, we performed additional analysis of predictability for LMD-related hard events and non-hard events: comparison of ROCs between iFR_LM_ and FFR_LM_ for (i) cardiac death and LM-MI; and (ii) LM-TLR (Fig. 5). The AUC was significantly higher for iFR_LM_ than FFR_LM_ for cardiac death and LM-MI (iFR_LM_: 0.80 [95% CI 0.59 to 0.99] vs. FFR_LM_: 0.42 [95% CI 0.13 to 0.70; *p* = 0.047]). Contrary, regarding LM-TLR, the AUCs were not statistically different between the two group (iFR_LM_: 0.72 [95% CI: 0.57 to 0.87] vs. FFR_LM_: 0.66 [95% CI: 0.49 to 0.83; *p* = 0.32). Namely, the overall difference of AUC between iFR_LM_ and FFR_LM_ in the deferral group displayed in Fig. 4 was mainly attributed to the different predictability for hard cardiovascular events (cardiac death and LM-MI).

## Discussion

From the largest international multicenter registry of LMD interrogated with coronary physiology, we were able to perform a direct comparison between iFR and FFR-guided revascularization decision-making in patients with stable CAD and LMD. Our main study findings are as follows (Graphic Abstract). Firstly, in line with previous reports [8–12], discordance between iFR/FFR was observed in ~20% of cases of LMD. Secondly, neither iFR nor FFR are predictive for MACE in LMD in whom revascularization was performed. Thirdly, and conversely, both iFR and FFR are predictive for MACE in LMD in whom revascularization was deferred. Lastly, iFR-guided deferral of LM-revascularization appeared to be safer than FFR-guided deferral.

### The role of coronary physiology in LMD according to the treatment strategy

The utility of risk stratification for patients with stable CAD in whom revascularization can be safely deferred is a well-established advantage of both iFR and FFR-guided revascularization decision-making [16–20]. Within the present study, this utility can apparently also be extended to LMD patients, as evidenced by the similar efficacy of both iFR_LM_ and FFR_LM_ to predict MACE in the LM-revascularization deferral group.

Once revascularization has been performed, however, in the LM-revascularization group of this study, baseline iFR_LM_ and FFR_LM_ values were not found to be predictors of MACE, as has been previously reported [21, 22]. This may suggest that the more important drivers of clinical outcomes in LM-revascularization are the judicious use of intracoronary physiology and imaging in PCI, and the routine use of internal mammary artery grafting during CABG [3, 23, 24].

### The Difference in clinical outcomes of LMD based on iFR- or FFR-guided strategy

As previously demonstrated [8–12], iFR/FFR discordance was observed approximately 20% of cases in the present study. Despite such physiological differences, the 3V FFRFRIENDS study reported that clinical outcomes were similar among the discordant population of non-LMD [25]. Conversely, a sub-analysis of the DEFNE-FLAIR study focusing only on LAD territory demonstrated that iFR-guided deferral was associated with significantly lower MACE as compared with FFR-guided deferral [26]. Regarding hard event, more recent reports focusing on the pooled 5 year mortality in the DEFINE-FLAIR and iFR-SWEDEHEART trials demonstrated significantly higher mortality in the iFR-arm [27, 28]. However, it was reported that the difference was not derived from the deferral group but from the revascularization group, which indicated that the value of epicardial coronary revascularization (*i.e.* PCI and CABG) might be substantially different for the iFR- or FFR-positive patients despite guideline-based interchangeable recommendation [29]. Furthermore, we have to recognize those trials fundamentally excluded LMD as well.

Accordingly, these conflicting reports provoke an even greater discussion in LMD because the LM stem subtends the largest myocardial territory in the coronary artery system [30]. A recent report from the DEFINE-LM registry demonstrated that outcomes in patients of physiologically significant (iFR≤0.89) LMD treated by medical therapy alone were not clinically acceptable, even with contemporary optimal medical therapy (MACE: ∼30% in 4 years) [3]. This study also suggested that a physiologically significant iFR should not be ignored regardless of reassuring results of other forms of ischemia assessment (*i.e.* negative findings for significant ischemia on non-invasive testing, FFR, or intravascular ultrasound).

This latter point is further supported by the current analysis, which also suggests a higher predictive power of iFR_LM_ in deferred patients with LMD (Fig. 4A and Supplemental Fig. S2). Furthermore, the direct comparison of clinical outcomes in the log-rank test for the groups classified by iFR_LM_ and FFR_LM_ showed significantly higher event rates in iFR_LM_-positive deferral group, though the sample size was relatively small to be conclusive (Supplemental Fig. S3). Accordingly, the present study demonstrates the importance of physiological assessment with iFR in the contemporary management of LMD (at least, prior to the FFR measurement). In our hypothetical insights, these differences in clinical outcomes might be attributed to the high rate of cardiovascular events of the deferred LMD as natural history [11]. Given that coronary microvascular dysfunction is better correlated with the lower iFR than lower FFR [31, 32], this was considered to be a potential mechanism for worse outcomes in the FFR-guided deferral patients in the previously reported sub-analysis of the DEFINE-FLAIR focusing on the LAD [26]. Similarly, in the most proximal disease of the LMD subtending the largest myocardial territory [30], the risk of cardiovascular events of the LMD with lower iFR and higher FFR would be enhanced by the influence of microvascular dysfunction, which would hamper the ability of maintaining cardiac system and could cause adverse cardiovascular events [33]. In fact, the overall difference of AUC between iFR_LM_ and FFR_LM_ in the deferral group was mainly attributed to the different predictability for hard cardiovascular events (cardiac death and LM-MI) in the present study (Fig. 4). However, as mentioned above, comprehensive understandings for the different outcomes between iFR and FFR could not be easily explained by the single study. Therefore, deeper investigations for this topic (which might include LMD and non-LMD as well as the assessment of coronary microvascular dysfunction) would be necessary for the current body of knowledge.

### Advantages of a multi-modal approach in LMD

Although our study did not demonstrate statistical significance between the physiological assessment with iFR_LM_ and the anatomical assessment with quantitative coronary angiography to predict MACE (Supplemental Fig. S4). Furthermore, the benefit of measuring FFR following iFR assessment was not supported (Supplemental Fig. S3). However, considering the small sample size of the study, and the known fact of the value of coronary physiology beyond coronary angiography [16–18], our study may not be conclusive in this regard. We believe a multi-modal approach is the best strategy to assess LMD, integrating anatomical (angiography and intracoronary imaging) and physiological (NHPR and FFR) data.

Indeed, the recently reported iLITRO-EPIC07 study suggested benefit following a combined usage approach of iFR, FFR, and intravascular ultrasound-guided revascularization decision-making [14]. Accordingly, dependence on any single modality as a truly definitive determinant of revascularization deferral in the LMD must be cautioned against. In order to further determine the true *‘state-of-the-art management of LMD’*, further study should independently validate the importance of a multimodality-based approach to guide revascularization decision-making (angiography, intracoronary imaging modality, NHPR and FFR, as well as non-invasive tests).

### Study limitations

The present study has several limitations. First, despite being the largest ever international multicenter registry of LMD interrogated with coronary physiology, the sample size was still relatively small. The small numbers of patients included might have affected the statistical significance or non-significance. Further studies should validate the current results in randomized controlled designs or larger registry studies.

Second, due to the non-randomized nature of this study, a potential for selection bias of iFR and FFR measurement for LMD must be considered. This was, however, an all-comers registry for stable CAD with LMD. The value of such a registry-based approach is that it reflects the patient population in real-world clinical practice.

Third, the reasons for treatment strategy chosen in each patient would undoubtedly have been multi-factorial. Indeed, revascularization was deferred in 81 patients despite physiological confirmation of myocardial ischemia (iFR $\le$ 0.89 or FFR $\le$ 0.80). Although the factors that were prioritized over the physiological cut-off values are provided in Supplemental Table S3, it was unclear how the respective reasons were weighted for decision-making in each patient.

Forth, due to the non-blinded nature of this study, a potential for selection bias of elective LM-TLR must be considered in the iFR/FFR discordant cases. The influence of preference of physician for specific modality (iFR or FFR) could not be excluded and thus, potentially, the decision for elective LM-TLR in the late phase could be biased as the discordant results were not blinded.

Fifth, there were 4.4% (12/275) patients with hemodialysis. There has been a great deal of discussion to use iFR or FFR for this specific patient subset because different cutoff values of intracoronary pressure indices might be appropriate (34–36). The current analysis could not provide any insights into physiology-guided management for patients with LMD and hemodialysis due to the limited number of such patients.

Finally, quantitative coronary angiography and physiologic tracing analysis were not performed at independent core laboratories.

## Conclusions

Within the sub-analysis of the DEFINE-LM registry, in LMD patients in whom physiological assessment was performed with both iFR and FFR, iFR had a higher predictive value for adverse cardiovascular events than FFR in the deferred LMD. Further comparative studies are warranted to evaluate the relative safety of iFR-guided vs. FFR-guided deferral strategies for LMD.

### Supplementary Information

Below is the link to the electronic supplementary material.

Supplementary file1 (DOCX 635 KB)

## Data Availability

The authors declare that all supporting data are available within the article and its online supplementary files.
