# Supplementary material for: Physiological Assessment with iFR prior to FFR Measurement in Left Main Disease
Source: Cardiovasc Interv Ther. 2024 Apr 20;39(3):241–51. doi: 10.1007/s12928-024-00989-4 (PMC11164744; doi:10.1007/s12928-024-00989-4)
Supplement: Supplementary file 1 — Supplementary file1 (DOCX 635 KB) [file 12928_2024_989_MOESM1_ESM.docx]

**Physiological Assessment with iFR prior to FFR Measurement**

**in Left Main Disease**

**- Supplemental Materials -**

**Supplemental Figures.**


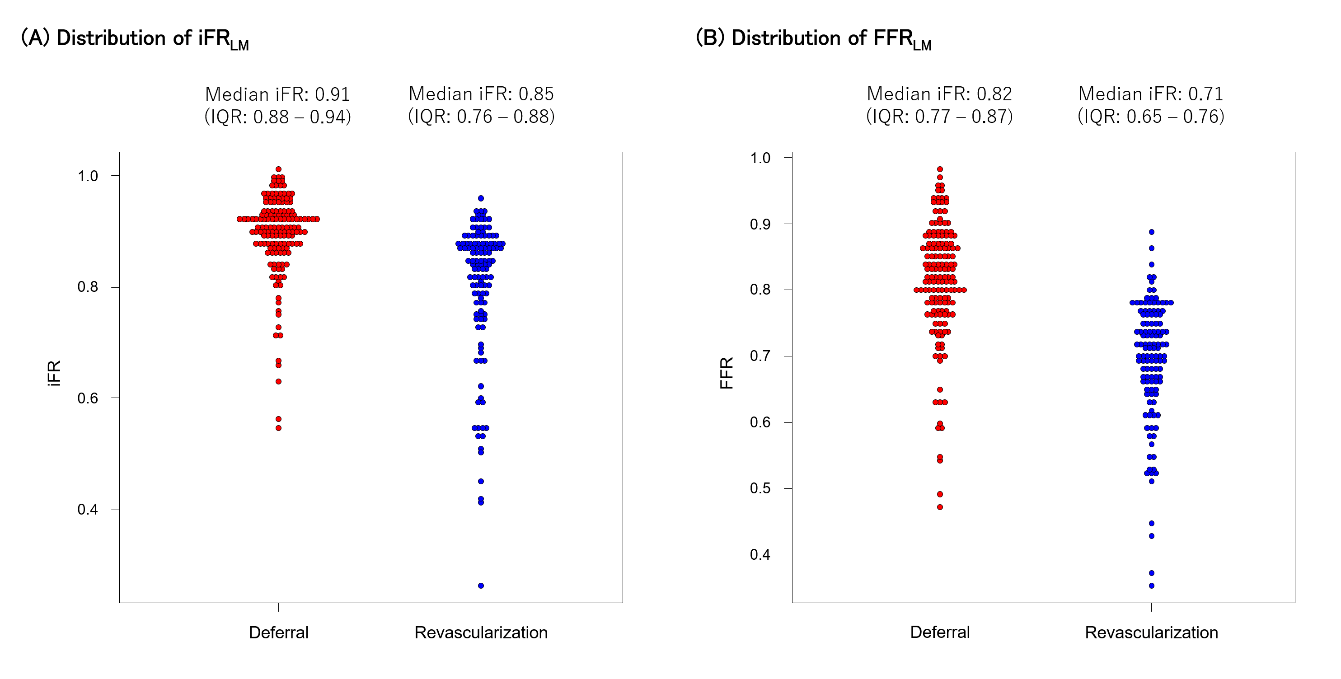
**Figure S1. Distributions of iFR_LM_ and FFR_LM_ Values according to the Treatment Strategy.**

FFR: fractional flow reserve; iFR: instantaneous wave-free ratio; IQR: interquartile range.

**Figure S2. Decision Curve Analysis for iFR_LM_ and FFR_LM_.**


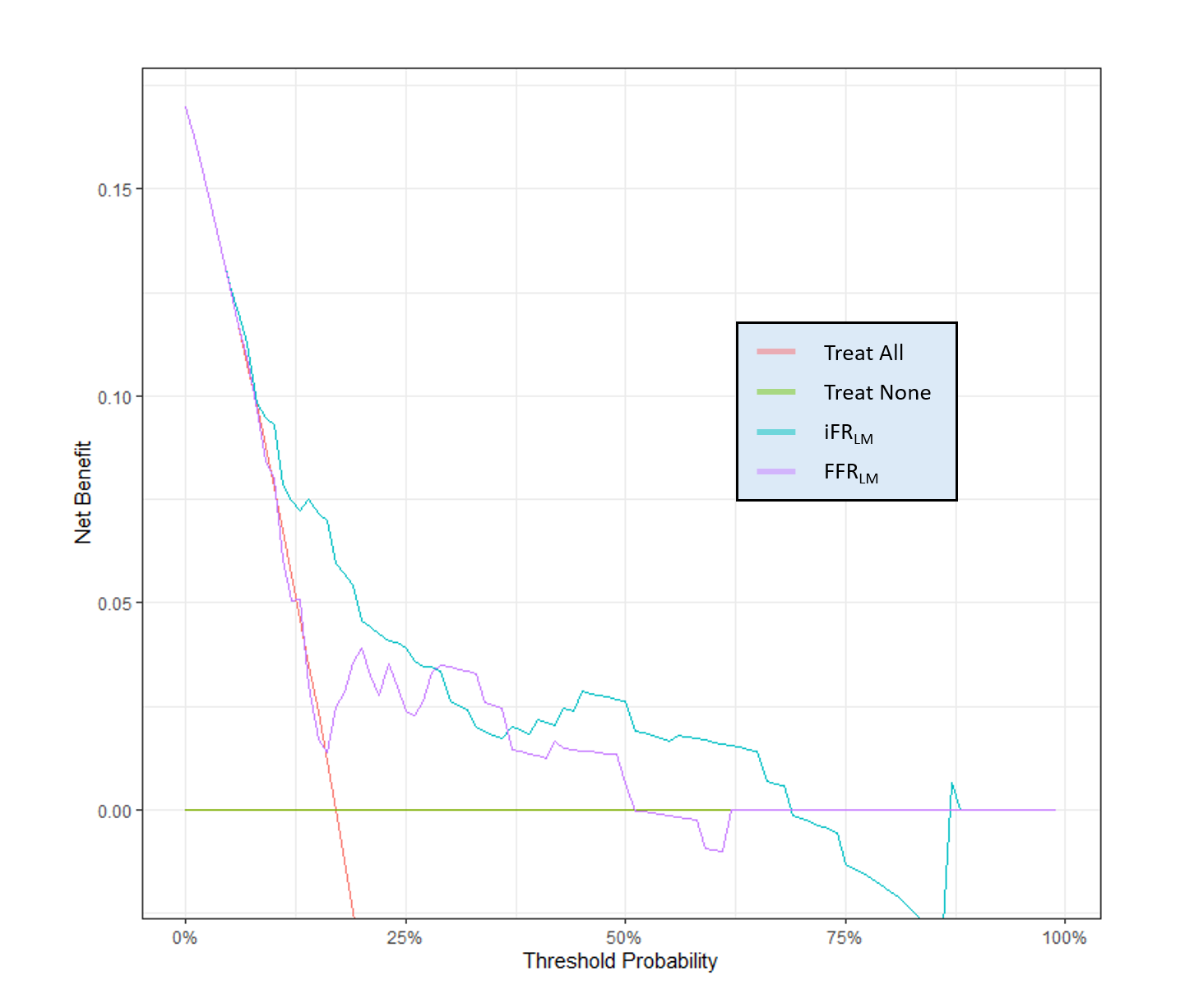


The decision curve analysis suggested that iFR_LM_ was more beneficial than FFR_LM_ for the prediction of MACE.

MACE: major adverse cardiovascular event. Other abbreviation as in Figure S1.


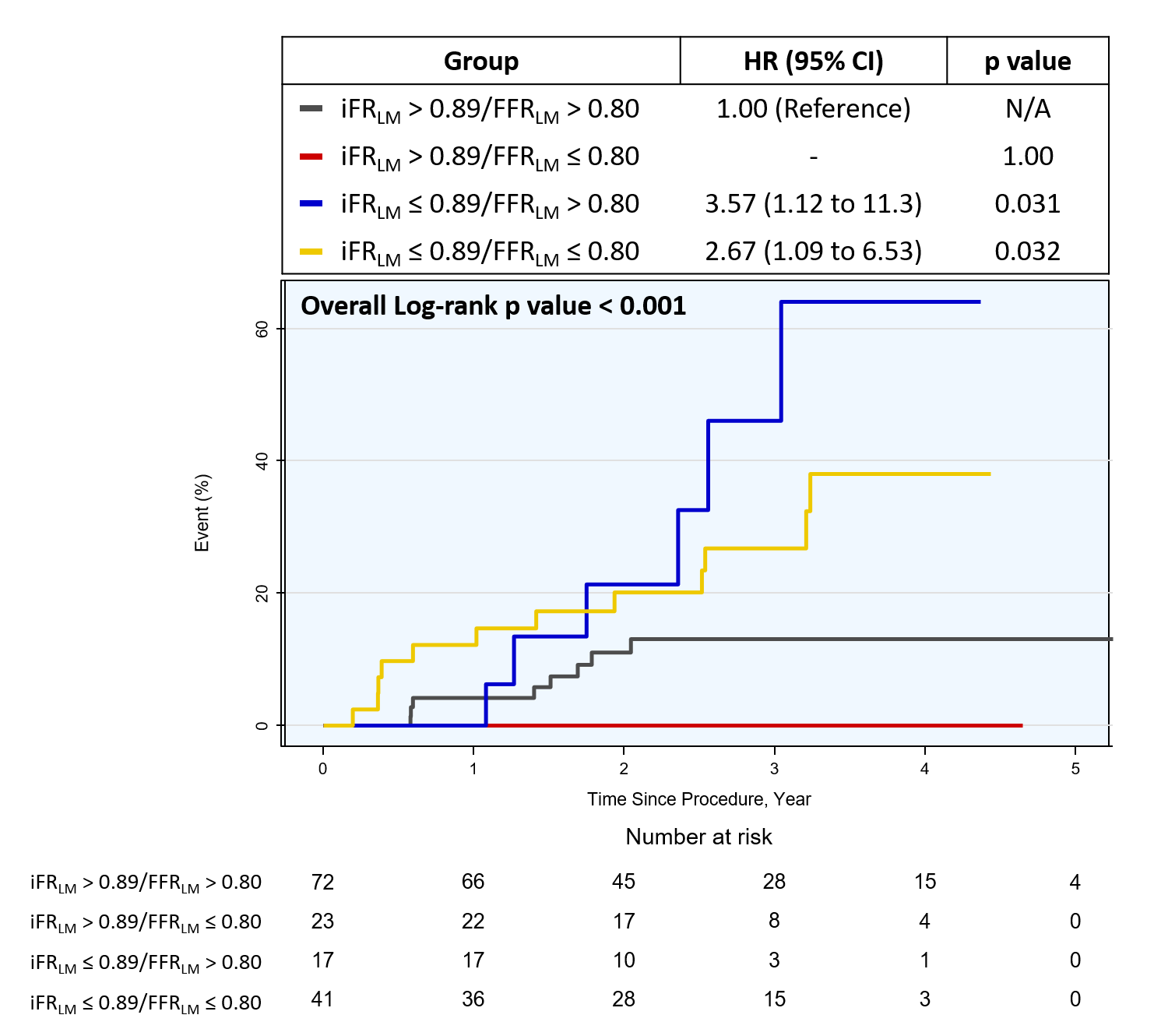
**Figure S3. Comparison of Clinical Outcomes of the Left Main Disease Classified by iFR_LM_ and FFR_LM_.**

In comparison to concordant normal results of iFR**_LM_** and FFR**_LM_** (iFR > 0.89 and FFR > 0.80), the rates of cardiovascular event were significantly higher in the classification of iFR**_LM_** $\leq$ 0.89 and FFR**_LM_** > 0.80 group and the concordant abnormal (iFR**_LM_** $\leq$ 0.89 and FFR**_LM_** $\leq$ 0.80) group. Conversely, the classification of iFR**_LM_** > 0.89 and FFR**_LM_** $\leq$ 0.80 group did not show any difference.

CI: confidence interval; HR: hazard ratio. Other abbreviation as in Figure S1.**Figure S4. iFR_LM_ vs. FFR_LM_ vs. QCA to Predict MACE in the Deferred Group.**


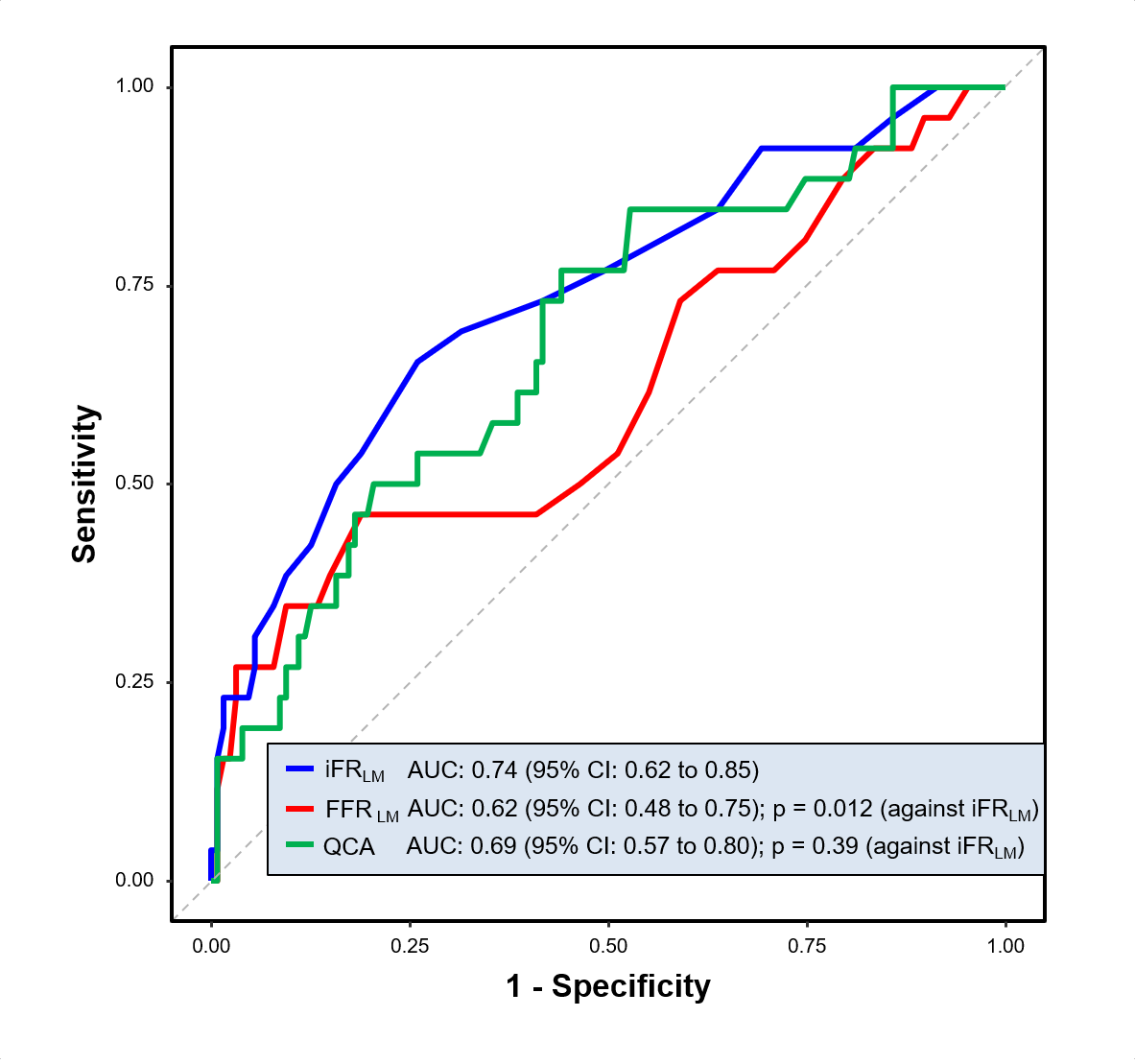


AUC: area under the curve; QCA: quantitative coronary angiography. Other abbreviation as in Figure S1 and S2.

**Supplemental Tables.**

**Table S1. Patient and Lesion Characteristics.**

| ***Patient characteristics* (n = 275)** | |
| --- | --- |
| Age, yrs | 67.9 ± 10.2 |
| Male | 215 (78.2) |
| Hypertension | 208 (75.6) |
| Dyslipidemia | 185 (67.3) |
| Diabetes mellitus | 102 (37.1) |
| Chronic kidney disease | 63 (22.9) |
| Current smoker | 91 (33.1) |
| Family history of CAD | 44 (16.0) |
| Previous MI | 77 (28.0) |
| ***Vessel and lesion characteristics*** | |
| ***Left main lesion type*** | |
| Ostial type | 68 (24.7) |
| Mid type | 62 (22.5) |
| Distal type | 220 (80.0) |
| ***Other diseased vessels*** | |
| No. of diseased vessels | |
| 0 | 47 (17.1) |
| 1 | 76 (27.6) |
| 2 | 93 (33.8) |
| 3 | 59 (21.5) |
| LAD | 176 (64.0) |
| LCx | 128 (46.5) |
| RCA | 135 (49.1) |
| With CTO | 28 (10.2) |
| SYNTAX Score | 19.6 ± 8.7 |
| ***Quantitative coronary angiography*** | |
| % Diameter stenosis, % | 45.2 ± 14.2 |
| Minimum lumen diameter, mm | 2.04 ± 0.67 |
| Reference diameter, mm | 3.80 ± 0.88 |
| Lesion length, mm | 13.3 ± 7.4 |
| ***Physiological stenosis severity*** | |
| iFR**_LM_** | 0.88 (0.82 - 0.92) |
| FFR**_LM_** | 0.77 (0.70 – 0.84) |

Values are mean $\pm$ SD, n (%), or median (interquartile range).

CAD: coronary artery disease; CTO: chronic total occlusion; FFR: fractional flow reserve; iFR: instantaneous wave-free ratio; LAD: left anterior descending artery; LCx: left circumflex artery; MI: myocardial infarction; MLD: minimum lumen diameter; RCA: right coronary artery; SYNTAX: Synergy Between Percutaneous Coronary Intervention With Taxus and Cardiac Surgery.

**Table S2. Cause of Non-Cardiac Death.**

| **Group** | **Age** | **Sex** | **Cause of Death** |
| --- | --- | --- | --- |
| Deferral | 68 | Male | Esophageal Cancer |
|  | 71 | Female | Colon Cancer |
|  | 73 | Female | Chronic Obstructive Pulmonary Disease |
|  | 84 | Female | Pneumonia |
|  | 86 | Male | Pneumonia |
| Revascularization | 68 | Male | Cerebral Hemorrhage |
|  | 86 | Male | Pneumonia |

**Table S3. The Prioritized Factors over the iFR or FFR Cut-off Values.**

| **Prioritized Factors over Physiological Values (n = 81)** | **n (%)** |
| --- | --- |
| Non-Invasive Tests | 19 (23.5) |
| Negative iFR | 23 (28.4) |
| Negative FFR | 32 (39.5) |
| Intravascular Ultrasound | 11 (13.6) |
| Diffuse Long Disease in the LAD | 0 (0.0) |
| Symptom | 5 (6.2) |
| Patient Preference | 4 (4.9) |
| Comorbidities | 3 (3.7) |

Values are n (%).

Abbreviation as in Table S1.
